# Mediating role of atherogenic lipoproteins in the relationship between liver fat and coronary artery calcification

**DOI:** 10.1038/s41598-023-39390-1

**Published:** 2023-08-14

**Authors:** Elias Björnson, Dimitrios Samaras, Martin Adiels, Joel Kullberg, Fredrik Bäckhed, Göran Bergström, Anders Gummesson

**Affiliations:** 1 Wallenberg Laboratory, Department of Molecular and Clinical Medicine and Sahlgrenska Center for Cardiovascular and Metabolic Research, Sahlgrenska University Hospital, University of Gothenburg, 413 45 Gothenburg, Sweden; 2 School of Public Health and Community Medicine, Institute of Medicine, University of Gothenburg, Gothenburg, Sweden; 3 Section of Radiology, Department of Surgical Sciences, Uppsala University, 752 37 Uppsala, Sweden; 4 Antaros Medical, 431 83 Mölndal, Sweden; 5 Department of Clinical Physiology, Sahlgrenska University Hospital, Region Västra Götaland, 413 45 Gothenburg, Sweden; 6 Department of Clinical Genetics, Sahlgrenska University Hospital, Region Västra Götaland, 413 45 Gothenburg, Sweden

**Keywords:** Cardiology, Gastroenterology, Endocrinology, Endocrine system and metabolic diseases

## Abstract

Non-alcoholic fatty liver disease (NAFLD) is associated with increased secretion of apoB-containing lipoproteins and increased risk of coronary heart disease (CHD). ApoB-containing lipoproteins include low-density lipoproteins (LDLs) and triglyceride-rich lipoproteins (TRLs); and since both LDLs and TRLs are causally related to CHD, they may mediate a portion of the increased risk of atherosclerosis seen in people with NAFLD. In a cohort of 4161 middle aged men and women, we performed mediation analysis in order to quantify the mediating effect of apoB-containing lipoproteins in the relationship between liver fat and atherosclerosis—as measured by coronary artery calcium score (CACS). We found plasma apoB to mediate 17.6% (95% CI 11–24) of the association between liver fat and CACS. Plasma triglycerides and TRL-cholesterol (both proximate measures of TRL particles) mediated 22.3% (95% CI 11–34) and 21.6% (95% CI 10–33) of the association respectively; whereas LDL-cholesterol mediated 5.4% (95% CI 2.0–9.4). In multivariable models, the mediating effect of TRL-cholesterol and plasma triglycerides showed, again, a higher degree of mediation than LDL-cholesterol, corroborating the results seen in the univariable models. In summary, we find around 20% of the association between liver fat and CACS to be mediated by apoB-containing lipoproteins. In addition, we find that TRLs mediate the majority of this effect whereas LDLs mediate a smaller effect. These results explain part of the observed CAD-risk burden for people with NAFLD and further suggest that TRL-lowering may be particularly beneficial to mitigate NAFLD-associated coronary artery disease risk.

## Introduction

The liver produces and secretes the majority of all apoB-containing lipoproteins in plasma, and this secretion is increased with increasing degrees of liver fat^1^. ApoB-containing lipoproteins in turn is the primary cause of coronary heart disease (CHD)^2,3^. Since liver fat has further been associated with ASCVD^4–6^ and with coronary artery calcification ^7,8^, the potential mediating role of apoB-containing lipoproteins in the possible causal chain between liver fat and ASCVD is subject for investigation. There are two broad sub-categories of apoB-containing lipoproteins; low-density lipoproteins (LDLs) and triglyceride-rich lipoproteins (TRLs). Both of these particles are atherogenic^9^, however TRLs may be more affected by increased liver fat than LDLs. The aim of the present study was to quantify the mediating role of apoB-containing lipoproteins in the association between liver fat and coronary artery calcification.

## Methods

### Cohorts

The present study is based on data from clinical studies conducted at the Sahlgrenska University Hospital, Gothenburg, Sweden. Participants (baseline characteristics presented in Table 1) were recruited to these studies from two community-based cohorts: (1) the Impaired Glucose Tolerance and Gut Microbiota Study (IGT-Microbiota study), which is a prospective observational study of Swedish people aged between 50 and 64 years. In the IGT-Microbiota study, more than 5000 men and women born in Sweden were screened to obtain a cohort of 1965 subjects with a range of glucose tolerance as assessed by an oral glucose tolerance test (OGTT)^10^, (2) The Gothenburg SCAPIS OGTT study, which consists of 3346 participants in the Gothenburg part of the Swedish CArdioPulmonary bioImage Study (SCAPIS) who underwent OGTT in addition to the SCAPIS protocol. SCAPIS is a prospective observational study with 30,154 individuals enrolled at ages between 50 and 64 years from a random sampling of the general Swedish population^11^.

**Table 1:** Baseline characteristics of the 4161 subjects stratified on NAFLD status.

NAFLD status	NO	YES	*P* value
	Median [IQR] or N(%)	Median [IQR] or N(%)	
N	3375	786	
Liver attenuation (HU)	59.0 [55, 62]	42.0 [34, 46.8]	< 0.001
CAC category			< 0.001
0	2170 (64.3)	371 (47.2)	
1–99	922 (27.3)	287 (36.5)	
100–399	216 (6.4)	88 (11.2)	
> 400	67 (2.0)	40 (5.1)	
Gender = male	1395 (41.3)	541 (68.8)	< 0.001
Age (y)	57.4 [53.6, 61.3]	58.6 [54.5, 61.9]	< 0.001
BMI (kg/m^2^)	25.6 [23.4, 28.1]	30.2 [27.5, 33.4]	< 0.001
Systolic BP (mm Hg)	120.5 [111, 132]	130 [120, 141]	< 0.001
Fasting glucose category			< 0.001
Diabetic	41 ( 1.2)	49 (6.4)	
Impaired	548 (16.3)	187 (24.5)	
Normal	2778 (82.5)	528 (69.1)	
HbA1c (mol/mol)	34.0 [32, 37]	36.0 [33, 39]	< 0.001
Plasma TG (mmol/l)	0.95 [0.72, 1.3]	1.50 [1.1, 2.0]	< 0.001
TRL-cholesterol (mmol/l)	0.10 [0, 0.30]	0.30 [0.1, 0.50]	< 0.001
Total cholesterol (mmol/l)	5.60 [5.0, 6.3]	5.60 [5.0, 6.3]	0.569
LDL-cholesterol (mmol/l)	3.70 [3.1, 4.3]	3.80 [3.3, 4.5]	< 0.001
HDL-cholesterol (mmol/l)	1.70 [1.4, 2.1]	1.30 [1.1, 1.6]	< 0.001
Non-HDL-cholesterol (mmol/l)	3.80 [3.2, 4.5]	4.20 [3.6, 4.9]	< 0.001
ApoB (g/l)	1.10 [0.92, 1.2]	1.20 [1.0, 1.4]	< 0.001
Smoke status			< 0.001
Never	1799 (53.3)	343 (43.6)	
Ex smoker	1272 (37.7)	337 (42.9)	
Current	304 (9.0)	106 (13.5)	

*P*-values are calculated using a chi-squared test for categorical values and the Mann–Whitney U test for continuous variables. *IQR:* Interquartile range.

In the two cohorts, imaging data on liver fat and coronary artery calcification were available in 1944 subjects in the IGT-Microbiota study and 2631 subjects in the Gothenburg SCAPIS OGTT cohort, leaving a total of 4575 subjects. Of this cohort, 322 subjects were excluded due to lipid lowering medication and a further set of 92 subjects were excluded due to missing lipid-and/or covariate measurements, leaving a total of 4161 to be included in the current study.

The studies are approved by the Ethical Review Board of University of Gothenburg, Sweden. All participants provided written informed consent. The study protocols conform to the ethical guidelines of the 1975 Declaration of Helsinki.

### Measurements

Liver fat and coronary artery calcification (CAC) was assessed by computed tomography (CT) scanning using a dual-source CT scanner equipped with a Stellar Detector (Siemens, Somatom Definition Flash, Siemens Medical Solution, Forchheim, Germany). CT is a well-established method to non-invasively quantify liver fat from liver attenuation values which are inversely correlated with liver fat content^12^. Mean liver attenuation was determined using an automatated liver segmentation algorithm^13^, based on a single CT slice positioned to cover both liver lobes**.** NAFLD was defined as liver attenuation < 50 Hounsfield Units (HU). CAC images were obtained using electrocardiogram-gated non-contrast cardiac CT imaging at 120 kV. All non-contrast image sets were reconstructed (B35f. HeartView medium CaScore) and CAC were identified and scored using the syngo.via calcium scoring software (Volume Wizard; Siemens) to obtain a CAC score according to Agatston. LDL-cholesterol (LDL-C) was directly measured using a homogenous assay (Roche Diagnostics). TRL-cholesterol (TRL-C) was defined as total cholesterol minus HDL cholesterol minus LDL cholesterol—hence this measure reflects the cholesterol content of TRL particles. ApoB was measured by an immunoturbidity, photometric method and represents total apoB (apoB100 + apoB48) in plasma. Body weight was measured with participants in light clothing, using calibrated scales, and the body mass index (BMI) was calculated by dividing the weight in kg by the square of the height in meters. Systolic and diastolic blood pressure (SBP, DBP) was registered in supine position and after 5 min of rest. The subjects fasted overnight (for at least 8 h) before the visits.

### Rationale for inclusion of lipid measurements in mediation models

Since LDLs and TRLs are the main atherogenic lipoproteins in most people, the aim of the current study was to quantify the mediatory effect of LDL- and TRL particle concentration (both added together and separate) on CACS. In the current study, we had access to the following variables: apoB, plasma TG, TRL-C, LDL-C, non-HDL-C and total cholesterol. The best proxy of LDL + TRL particle concentration is total plasma apoB (which quantifies the particle concentration of LDL + TRL + Lp(a)) and the second-best proxy is non-HDL-C (which quantifies the cholesterol content of the LDL- and the TRL fraction). The closest measure of TRL particle concentration is TRL-C (cholesterol content of TRL) and plasma TG (triglycerides in plasma are mostly constituted by TRL-TG). The closest proxy of LDL particle concentration in the current study is LDL-C.

Therefore, the main variable to assess the total mediating effect of LDL + TRL particles is plasma apoB; and the main variables used to assess the mediating effect of LDL and TRL separately are LDL-C and TRL-C/plasma TG, respectively. For completeness, results for non-HDL-C and total cholesterol is also presented in the current report.

### Statistical analysis

Mediation analysis quantifies the relationship between an exposure and an outcome, via the mediation of an intermediating variable^14^. The analysis requires two regression models. The first model regresses the exposure on the mediator, whereas the second model regresses the exposure and the mediator on the outcome. Both models should include confounders of the association as covariates. The effect of the exposure on the outcome through the mediator can be estimated by multiplying the effect of the exposure on the mediator with the effect of the mediator on the outcome, while adjusting for the exposure variable. Mediation analysis was performed in R (version 4.0.2) using the *mediation* package. The analyses quantified the effect of liver attenuation (modelled as a continuous variable) on CACS (modelled as 0, 1–99, 100–399, and > 400) through either plasma TG, TRL-C, LDL-C, total plasma cholesterol, and non-HDL-cholesterol (non-HDL-C). In the regression models, age and gender were used as covariates. In addition to these covariates, four more models with different sets of covariates were used as sensitivity analyses (for further description, see also Supplementary Fig. 1). These models included BMI, systolic blood pressure or smoking status in addition to age and gender, as well as a model including all five covariates. Smoking and systolic blood pressure were chosen as these may be causal factors for CAC, and may correlate with liver fat. In order to quantify the independent effects of TRLs from LDLs, multivariable models were performed using the *lavaan* package in R. Two multivariable models were constructed; one model including TG and LDL-C and one model including TRL-C and LDL-C. These two multivariable models were chosen because plasma TG and TRL-C are the closest proxies for TRL particle abundance, whereas LDL-C reflects LDL particle abundance. Logistic regression analysis was performed using the function *glm* in R, in order to quantify the relationship between NAFLD-status and (a positive) CAC score.

## Results

**Figure 1:**
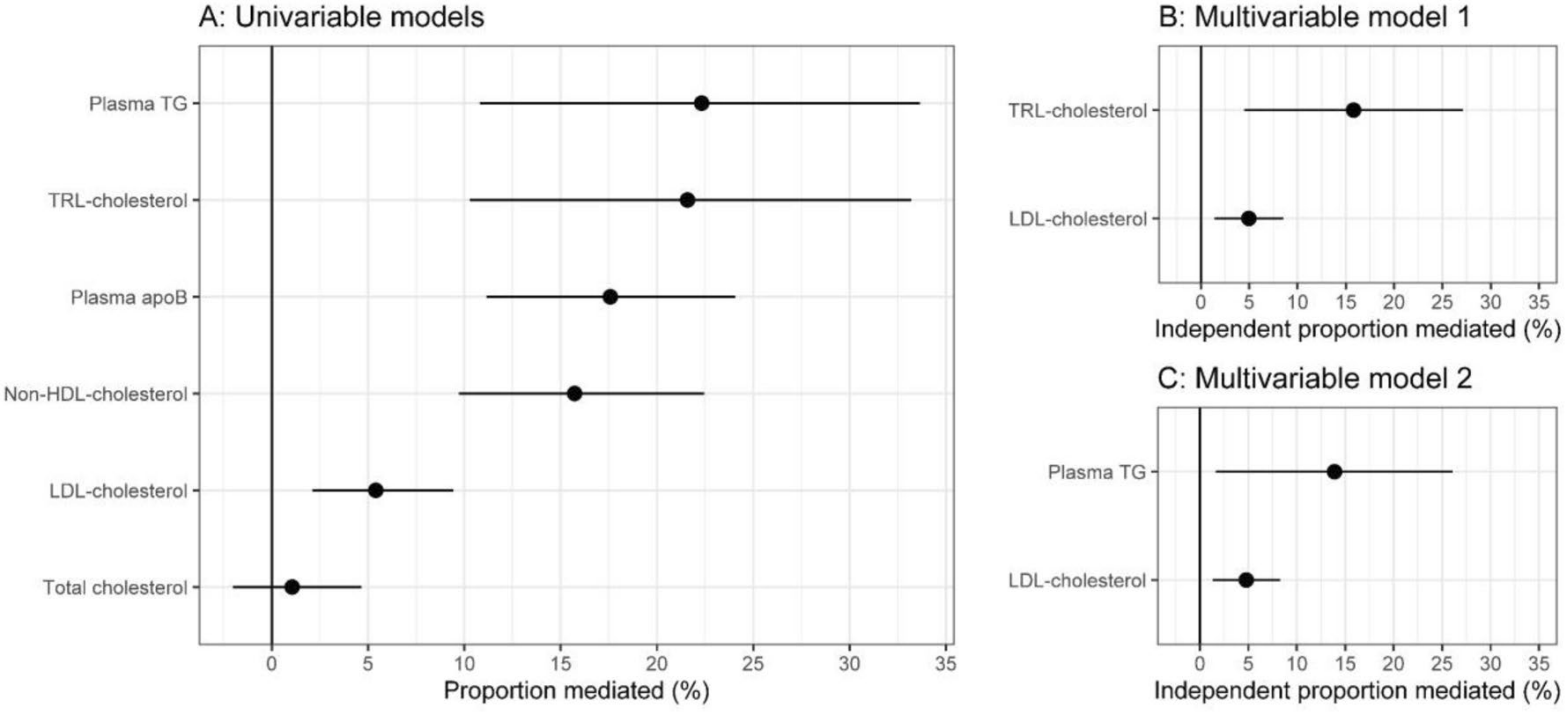
(**A**) Proportion of liver fat-to-CACS relationship mediated by: Plasma TG, TRL-C, ApoB, non-HDL-C, LDL-C and total cholesterol. Each variable is included in separate mediation models and thus do not account for intercorrelations between variables. (**B**) Multivariable model including TRL-C and LDL-C showing that both variables have an independent mediating effect with a point estimate of around 5% for LDL-C and around 15% for TRL-C. (**C**) Multivariable model including plasma TG and LDL-C showing similar results as in B. All effect-estimates are adjusted for age and gender, error bars indicate 95% CI.

Of the 4161 participants, 46.5% were male. Mean age was 56.7 years and 18.9% were classified as having NAFLD (Table 1). Of all participants, 61% had a zero CAC score. Without adjusting for lipoproteins, we found an association between liver fat and a positive CAC score. The odds ratio for a model including age and gender as covariates was 1.16 (95% CI 1.09–1.25) per 1 SD increased liver fat. In the mediation analyses, TG and TRL-C was found to mediate 22.3% (95% CI 11–34) and 21.6% (95% CI 10–33) of this association between liver fat and CACS respectively (Fig. 1, Table 2). The point estimates changed marginally when including more or less covariates, although in models including BMI, the mediated proportion dropped to around 13% for TRL-C whereas it did not change considerably for TG (Table 2). In a model with only age and gender as covariates, the point estimate for LDL-C was significant at around 5.4% (95% CI 2.0–9.4). In models including BMI as a covariate, LDL-C was not significant, whereas in models not including BMI it remained at around 5–6% (Table 2).

**Table 2:** Mediating effect (as percent of total effect) of plasma lipids/lipoproteins in the relationship between NAFLD and CACS. Point estimates are shown with 95% confidence intervals in parenthesis. Data in column one is the same data as shown in Fig. 1, whereas the rest of the models including different sets of covariates are shown in column 2–5. Univariable models are performed for all plasma-lipid related variables whereas the multivariable models show the independent mediating effect of LDL-C in relation to TRL-C (model 1) and to plasma TG (model 2).

Variable	Covariates				
	Age, gender	Age, gender plus BMI	Age, gender plus SBP	Age, gender plus smoking	All covariates
Univariable models					
Plasma TG	22.3 (11–34)	19 (6.1–32)	23.4 (8.2–39)	23.6 (10–36)	17.7 (1.7–35)
TRL-C	21.6 (10–33)	13.1 (3.9–23)	19.4 (6.3–34)	19.3 (6.6–32)	12.5 (− 0.28–26)
Plasma apoB	17.6 (11–24)	13 (5.6–22)	19.2 (12–27)	18.3 (12–26)	14.8 (5.5–28)
Non-HDL-C	15.7 (10–22)	8.72 (2.5–16)	16.4 (9.8–24)	16.2 (10–23)	10.4 (1.7–20)
LDL-C	5.4 (2.0–9.4)	− 0.34 (− 5.7–5.2)	5.37 (1.2–11)	5.74 (1.8–10)	− 1.32 (− 9.5–6.8)
Total cholesterol	1.1 (− 2.0–4.6)	− 0.23 (− 6.3–5.1)	0.221 (− 3.7–3.9)	0.774 (− 2.5–4.2)	− 1.49 (− 9.6–5.8)
Multivariable model 1					
TRL-C	15.8 (4.5–27)	12.4 (1.2–24)	15.9 (2.4–30)	15.1 (2.7–28)	12.1 (− 2.4–27)
LDL-C	5.0 (1.4–8.5)	− 0.1 (− 5.5–5.2)	4.9 (0.7–9)	5.3 (1.4–9.3)	− 1.1 (− 8.3–6.2)
Multivariable model 2					
Plasma TG	13.9 (1.6–26)	12.6 (− 1.2–26)	13.6 (− 1.1–28)	12.6 (− 0.9–26)	10.9 (− 6.7–29)
LDL-C	4.8 (1.3–8.3)	− 0.1 (− 5.3–5)	4.7 (0.6–8.8)	5.2 (1.3–9.1)	− 1.1 (− 8.2–6.1)

Plasma apoB and non-HDL-C showed intermediate effects at 17.6% (95% CI 11–24) and 15.7% (95% CI 10–22) respectively. Total cholesterol did not show a mediating effect. Lastly, to account for possible intercorrelations between LDLs and TRLs, we performed multivariable mediation analysis with TRL-C or TG together with LDL-C in two separate models (Fig. 1, Table 2). Using age and gender as covariates, we found TRL-C and TG to mediate 15.8% (95% CI 4.5–27) and 13.9% (95% CI 1.6–26) of the association between liver fat and CACS respectively. LDL-C mediated 5% (95% CI 1.4–8.5) when compared with TRL-C and 4.8% (95% CI 1.3–8.3) when compared with plasma TG.

## Discussion

Here, in a cohort of over four thousand middle aged men and women with CT-assessed liver fat and coronary calcium, we performed mediation analyses with the aim of quantifying the plausible mediating effect of apoB-containing lipoproteins in the relationship between liver fat and CACS. Previous research has investigated the association between NAFLD and CHD outcomes, and between NAFLD and coronary artery disease. To our knowledge, no previous lipoprotein-centric mediation-analyses have been performed in this context.

Firstly, we corroborate previous findings of an association between liver fat and CACS. We observed an increased risk of coronary calcification with increased liver fat in the main model. When adjusting for other covariates (systolic blood pressure, smoking and HbA1c) the estimates did not substantially change and when adjusting for the effect of BMI, the odds ratio fell and was borderline statistically significant (data not shown). However, controlled dietary trials show that increases in body weight (as a result of hypercaloric intake) is likely a causal factor for increased liver fat^15^; thus throughout the models in the current paper, using BMI as a covariate likely constitutes over-adjustment.

Next, in the mediation analyses, we find that plasma apoB (as a measure of all atherogenic lipoproteins) mediates around 15–20% of the association between liver fat and CACS. Since apoB includes both LDL particles and TRL particles, this estimate is an aggregate of these two lipoprotein species. Therefore, we performed mediation analyses on TRL-C/TG on one hand and on LDL-C on the other hand. As shown in Fig. 1, TG/TRL-C was found to mediate around 20% of the association, whereas LDL-C mediated around 5% in univariable models. Since lipoproteins show a degree of intercorrelation, the LDL-C estimate may partly include the TG or TRL-C estimate or vice versa. Therefore, we performed multivariable models in addition to the univariable models. When including both TRL-C/TG and LDL-C in multivariable models, the TG/TRL-C estimates dropped to around 15% whereas LDL-C remained at around 5%. Taken together, these results are in line with the notion that increased liver fat results in a somewhat larger effect on triglyceride-rich lipoproteins, and a lesser effect on low-density lipoproteins—as opposed to a uniform effect across the apoB spectrum. These results thus highlight a particular relationship between liver fat and triglyceride-rich lipoprotein metabolism. There are currently limited options for lowering triglyceride-rich lipoproteins, but interventions leading to liver fat reductions (through e.g. weight loss) may result in lowered ASCVD risk through lowering of TRLs. Reducing residual cardiovascular risk is important in general, and not least in the NAFLD patient population.

One limitation of the current study is the use of coronary calcium as outcome variable. CACS does not automatically translate into events which limits the interpretation and extrapolation of the current results. However, CACS is a close-to direct measure of coronary artery disease itself, and hence provide a good basis for evaluating the mediating effect of lipoproteins on coronary artery disease status. Another limiting factor for extrapolation of the results is that the current cohort may be enriched in participants of worse metabolic health than the general population. However, NAFLD generally co-varies with poorer metabolic health, making the results more likely relevant to this particular population. Thirdly, there are limitations in the mediation analysis framework, most prominently, it is difficult to construct models that represents the underlying causal structure and which fully accounts for all possible confounding. Lastly, TRLs are constituted by both intestinally-derived (apoB48-containing) lipoproteins and hepatically-derived (apoB100-containing) lipoproteins but since we did not quantify apoB48 separately, the intestinally-derived lipoproteins will be part of our estimation of TG/TRL-C.

In conclusion, we find apoB-containing lipoproteins to mediate around 20% of the relationship between liver fat and coronary calcium in a cohort of over four thousand middle aged men and women. Low-density lipoproteins mediate a minor part, whereas triglyceride-rich lipoproteins constitute the majority of the mediating effect.

### Supplementary Information

Supplementary Figure 1.

## Data Availability

Data may be available upon request by contacting corresponding author.
