# Supplementary material for: Mediating role of atherogenic lipoproteins in the relationship between liver fat and coronary artery calcification
Source: Sci Rep. 2023 Aug 14;13:13217. doi: 10.1038/s41598-023-39390-1 (PMC10425432; doi:10.1038/s41598-023-39390-1)
Supplement: Supplementary file 1 — Supplementary Figure 1. [file 41598_2023_39390_MOESM1_ESM.pdf]

Supplementary Material

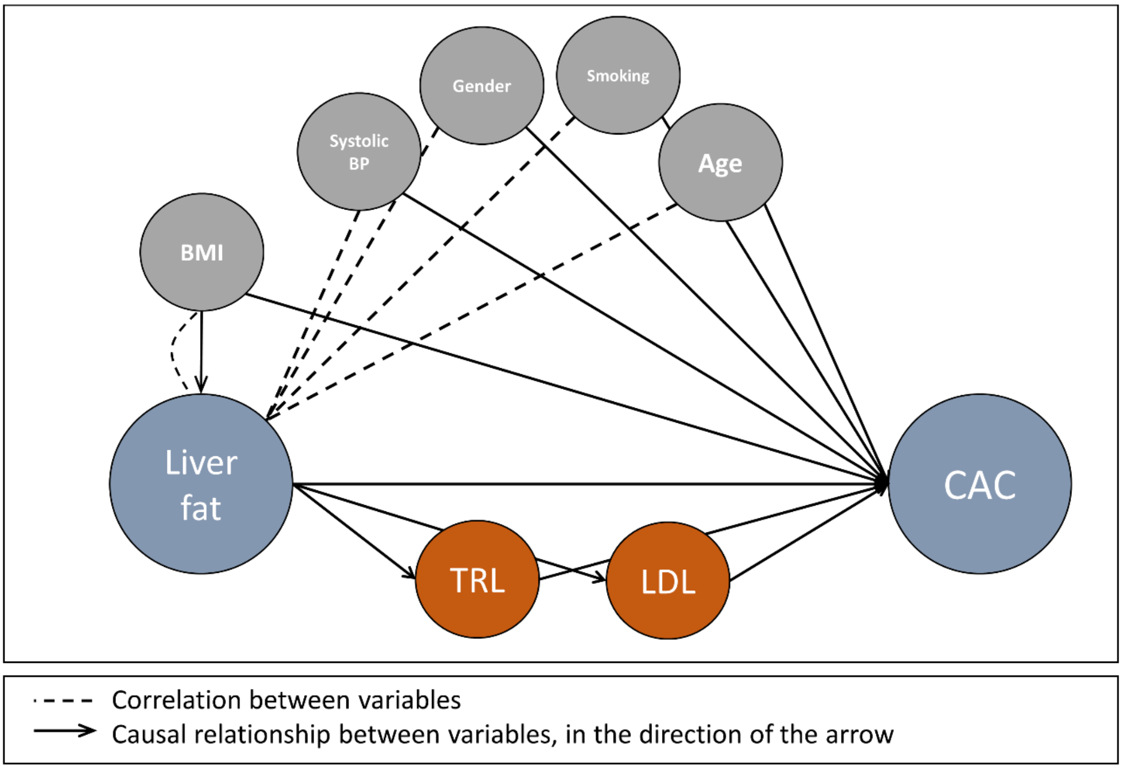

Supplementary Figure 1. Layout of the plausible relationship between the variables as hypothesized in the current investigation. The relationship between liver fat and coronary artery calcium (CAC) is indicated as a direct effect, and effects that is mediated by LDLs and TRLs separately. The covariates considered are highlighted in gray and include BMI, systolic blood pressure, gender, smoking and age. Covariates that may correlate with liver fat (indicated by dashed line) and constitute causal factors (indicated by solid arrow) for CAC should be included as covariates in the mediation analyses. BMI may also be considered a causal factor for liver fat (which would make it unsuitable as covariate) but was nevertheless included because of uncertainty of the exact relationship.
